# Optimizing Protocols for Arabidopsis Shoot and Root Protoplast Cultivation

**DOI:** 10.3390/plants10020375

**Published:** 2021-02-15

**Authors:** Taras Pasternak, Ivan A. Paponov, Serhii Kondratenko

**Affiliations:** 1 Institute of Biology II/Molecular Plant Physiology, Centre for BioSystems Analysis, BIOSS Centre for Biological Signalling Studies University of Freiburg, 79104 Freiburg, Germany; 2 Department of Food Science, Aarhus University, 8200 Aarhus, Denmark; ivpa@food.au.dk; 3 Institute of Vegetables and Melon Growing of National Academy of Agricultural Sciences of Ukraine, 62478 Kharkiv, Ukraine

**Keywords:** *Arabidopsis thaliana*, protoplasts, reprograming, auxin

## Abstract

Procedures for the direct regeneration of entire plants from a shoot and root protoplasts of *Arabidopsis thaliana* have been optimized. The culture media for protoplast donor-plant cultivation and protoplast culture have been adjusted for optimal plant growth, plating efficiency, and promotion of shoot regeneration. Protocols have been established for the detection of all three steps in plant regeneration: (i) chromatin relaxation and activation of auxin biosynthesis, (ii) cell cycle progression, and (iii) conversion of cell-cycle active cells to totipotent ones. The competence for cell division was detected by DNA replication events and required high cell density and high concentrations of the auxinic compound 2,4-D. Cell cycle activity and globular structure formation, with subsequent shoot induction, were detected microscopically and by labeling with fluorescent dye Rhodamine123. The qPCR results demonstrated significantly upregulated expression of the genes responsible for nuclear reorganization, auxin responses, and auxin biosynthesis during the early stage of cell reprogramming. We further optimized cell reprogramming with this protocol by applying glutathione (GSH), which increases the sensitivity of isolated mesophyll protoplasts to cell cycle activation by auxin. The developed protocol allows us to investigate the molecular mechanism of the de-differentiation of somatic plant cells.

## 1. Introduction

*Arabidopsis thaliana* represents the best-studied model among higher plants. Numerous investigations have been performed to characterize the fundamental mechanisms underlying totipotency, pluripotency, and nuclear reprogramming, including epigenetic regulation (for a review see Birnbaum, K. D., & Roudier, F. (2017) [1], and the use of Arabidopsis has added significant new insights into these mechanisms. However, the heterogeneity of the cells in Arabidopsis tissues complicates any attempts to track the developmental lineage and limits the application of state-of-the-art gene expression methods, such as microarrays and proteomics, which require a population of homogeneous cells. These limitations have been described recently and have been extended to include the additional complexity presented by individual organs that contain cells with different responses to stimuli and different regenerative potentials [2]. 

Specifically, only a restricted number of cells in the plant body maintain their full regenerative potential, while other cells very rapidly lose this capacity because of their rapid differentiation, polyploidization, and an inability to enter into the cell cycle. 

One way to overcome these limitations’ complexity is to use protoplasts, which represent a relatively homogenous plant cell population that lacks cell-to-cell communication and can be easily isolated. The first protoplast isolation and culture experiments in Arabidopsis were performed 45 years ago [3]. Subsequently, Damm and Willmitzer [4] reported successful shoot regeneration from leaf protoplasts, but the plating efficiency was exceptionally low. Several other similar protocols have been advanced for Arabidopsis plant regeneration from protoplasts [5,6]. However, the efficiency remains low, and shoot formation requires a prolonged culture period.

Recently, Chupeau et al. [7] reported a plating efficiency up to 30 to 50% of the initially plated shoot-derived protoplasts isolated from three-week-old seedlings, with the first shoot formation appearing after two months from the moment of protoplast isolation. However, the plant regeneration was not direct and was achieved only by subsequent de novo induction of shoot formation from a micro-colony.

The optimization of an efficient and reproducible protocol for the conversion of homogenous populations of differentiated somatic cells into totipotent cells is highly desirable for investigations into plant development, physiology, biochemistry, and molecular genetics. At present, no reports have been published on the isolation and cultivation of protoplasts derived from Arabidopsis roots. A literature search uncovered only one report of protoplast isolation from auxin-activated Arabidopsis root culture, which is different from native roots and can be considered rather as callus [8]. Thus, further development of protocols for protoplast isolation from native roots is still required. 

Here we describe a robust, optimized system for the isolation and cultivation of protoplasts from Arabidopsis shoots and roots. This protocol allows us to regenerate shoots in 30–40 days after protoplast isolation and to obtain seeds from the regenerated plants within the next two months [9].

## 2. Results and Discussion 

### 2.1. Shoot-Derived Protoplasts

#### 2.1.1. Starting Material 

The protoplast plating efficiency (number of cell divisions per number of plated protoplasts) is strongly dependent on the donor plant's growing conditions, particularly the medium composition and light conditions [10]. This point is especially true for Arabidopsis, which is a small plant with a short vegetative period that is accompanied by the rapid exit of leaf cells from the cell cycle [11], as well as rapid DNA reduplication that is promoted by high nitrate concentrations [12]. We tested more than 100 different nutrient combinations for Arabidopsis and determined that Optimized Arabidopsis medium 1 (TK1) [9] with an NPK ratio of 5:1:3, which is close to the classical Hewitt nutrient solution ratio [13,14] is optimal for in vitro plant culture. 

We directly compared the TK1 and Arabidopsis Medium (AM, ½ MS) media for effects on root growth, under the assumption that the traits of root growth and organization of the root meristems are sensitive to the mineral nutrition provided by the growth media. We found that plants grown on TK1 medium formed longer and more organized root meristems with significantly fewer abnormalities than were observed in plants cultivated in AM medium [15], suggesting that the TK1 medium was a more optimal choice [9]. 

Arabidopsis plants cultivated in TK1 medium generated good starting material for isolation of protoplasts that showed fewer endocycle rounds and regular ploidy. We also found that the addition of a low concentration of bacto-tryptone was essential for optimal plant growth because this addition prevented the formation of an insoluble pellet [16], thereby ensuring that all crucial nutrient elements remained in the available form (Table 1). 

#### 2.1.2. Protoplast Sources

Mesophyll protoplasts can be easy isolated from fully expanded leaves of most dicotyledonous plant species grown in vitro. However, this isolation is quite a challenging task when using Arabidopsis grow in vitro because of the very small plant size and rapid cell differentiation.

Protoplasts can also be isolated from cotyledons and hypocotyls, but only at certain developmental stages. In Arabidopsis grown in vitro, the protoplasts should be isolated before the cotyledon/hypocotyl cells pass the DNA re-duplication stage and the accompanying histone/DNA methylation that occurs four days after germination [17,18]. 

#### 2.1.3. Protoplasts Isolation Procedure 

The first step in protoplast isolation is transplanting the cells from the shoot tissue into the isolation buffer, where the shoot tissue is incubated for a prolonged time with cellulolytic enzymes. During this step, chromatin condensation [19] and alteration in the physiological features of the mesophyll cells occurred (for review, see [2]). 

In our current protocol, we consider the influence of most factors that can reduce apoptosis in protoplasts. The first factor is the minimization of the number of damaged cells, as these release proteases and nucleases into the isolation buffer. This minimization can be achieved by gently cutting the shoot into 0.5 mm thick strips with an exclusively new scalpel or a new razor blade. This strip thickness maintains many of the cells in an undamaged condition while still allowing efficient penetration of the cellulolytic enzyme into cell walls for digestion. By contrast, chopping off the shoots leads to a large number of damaged cells and must be avoided. 

The second factor is ensuring an appropriate ratio between tissue amount and enzyme volume. Typically, for a single isolation procedure, we use one 120 mm square Petri dish containing 100–120 seedlings with shoot fresh weights of around 250–300 mg. This amount of tissue has approximately 6 million cells and digestion occurred in 3-4 ml of enzyme solution (tissue: enzyme solution ratio is 1:10–1:15). Increasing enzyme volume does not lead to increased cell wall digestion efficiency; therefore, we recommend using 1:10 ratio. 

The third factor for reducing apoptosis is washing of the cut shoot strips to remove the DNAse/proteases eluted from damaged cells. Pre-warming of the cellulolytic enzyme’s solution to +55°C also reduces DNAse/protease activities during the digestion procedure [20]. Cutting shoots directly in enzyme solution without further washing demonstrated significantly decreased protoplasts yield.

The fourth factor is a replacement of the isolation buffer containing the cellulolytic enzymes with a W5m (modified W5 medium, see M&M [21]) solution after the cell wall digestion step. This replacement significantly increases the release of cells from the digested leaf strips and increases protoplasts viability. In the isolation buffer, as well as in the W5m solution, we used Ca(NO_3_)_2_ instead of CaCl_2_. This replacement is essential because it prevents further vacuolar acidification and cell expansion.

Altogether, we compared the yield of viable cells isolated after adjusting all of these factors with cell isolation after the standard protocol. Using the improved method, we were able to isolate 1.5 × 10^6^ ± 0.2 × 10^6^ viable protoplasts to compare with 0.5 × 10^6^ ± 0.1 × 10^6^ viable protoplasts isolated with the standard method from 250 mg of the shoot (N = 4).

#### 2.1.4. Medium for Protoplast Cultivation 

The composition of the TK30 medium (Table 2) is similar to the medium used for plant growth, apart from the addition of glucose, mannitol, and sucrose for osmotic adjustment, and it satisfies the general plant cell requirements for mineral nutrition. TK30 has an optimized N:P ratio of 6.22:1 (versus 27:1 for the Kao and Michayluk medium [22]) and a reduced concentration of Cl^−^ (0.6 mM). The higher Cl^−^ content of other media, such as Kao and Michayluk ([22] and PCA [23], at 12 mM and 6.12 mM Cl^−^, respectively, increase Cl^−^ sequestration in the vacuole. This Cl^−^ sequestration could contribute to increased cell size [24] due to the increased size of vacuole with changes typical for lytic vacuole [25]. This process is the reverse of what is observed during dedifferentiation when the acidic lytic vacuole is converted to a protein storage vacuole with alkaline properties [26]. This Cl^−^-enhanced cell expansion induces cell differentiation, and it also requires a high osmotic pressure in the medium to prevent cell damage, thereby reducing cell viability. 

We have also adjusted the Zn^2+^/Cu^2+^ ratio to 3:1 instead of the 20:1 used in the Kao and Michayluk medium. Zinc (Zn^2+^) is one of the most abundant metals in living organisms [27] and has fundamental roles in diverse physiological processes, including carbohydrate, lipid, and nucleic acid metabolism and protein synthesis. Cu is another vital metal involved in the regulation of many enzymes, including Cu^2+^/Zn^2+^ superoxide dismutase (SOD), cytochrome c oxidase, amine oxidase, laccase, plastocyanin, and polyphenol oxidase. Cu^2+^ and Zn^2+^ interact with each other in many reactions, so optimal ratios are required. All these changes allow us to increase plating efficiency from 17% on the Kao and Michayluck medium to 40–50% in this protocol (Figure S1).

As for growth-regulator adjustment, different concentrations have been tested. In combination with TK30 ion balance, 2 µM 2,4-D gave the highest proliferation ratio (40.6 ± 1.79%), while further increase of 2,4-D concentration was toxic and inhibited proliferation. 

#### 2.1.5. Osmotic Pressure Adjustment for Selecting Cells with High Proliferative Capacity 

The critical condition for cell reprogramming is a regular ploidy level in donor cells. Nicotiana [28] and Medicago cells [29] have regular ploidy; however, Arabidopsis cells become polyploid due to DNA endoreplication in leaf cells [18]. Polyploid protoplasts are viable when given an optimal nutrient balance but they never reprogram because of the loss of DNA fragments. We selected cells with regular ploidy using an osmotic pressure adjustment approach. This approach allows the selection of cells with a high proliferative capacity and aids in maintaining cell viability. 

This approach is based on the different properties shown by cells at different ages. During differentiation *in planta*, the cell volume continuously increases due to an increase in vacuole size in response to increasing internal osmotic pressure (via VPPase/V-ATPase activity). After isolation, protoplasts still retain the majority of the physiological parameters of the donor cells [30]. Therefore, protoplasts from tissues of older physiological age have a higher internal osmotic pressure than is observed in protoplasts from younger, juvenile tissue. Taking into account this difference, we used a combination of glucose (0.4 M), mannitol (0.1 M), and sucrose (0.05 M) (TK30 medium) to ensure the integrity of competent cells during cultivation. We further improved the yield of competent cells by incubating the protoplasts in 0.45 M glucose (1–2 h), followed by an additional purification step in a sucrose gradient to remove damaged cells. The importance of the correct selection of the osmotic pressure of the nutrient medium for the cultivation of protoplasts of higher plants at the initial stage of cultivation was confirmed in our previous work [31]. 

Optimization of osmotic pressure was performed for the incubation of the isolated protoplasts on the medium supplemented with 0.4 M, 0.5 and 0.55 M osmolytes. Only 10% of the cell survived after the incubation in a 0.4 M osmotic medium, and under these conditions, no cell division was detected. While increasing osmotic concentration to 0.55 M led to 99% cell viability after the first 24 h and maximal yield for cell division (37.9 ± 1.8%) after 96–120 h. 

#### 2.1.6. Organic Acid Supplementation and Protoplast Plating Density 

One of the most important components of the protoplast culture medium is the organic acid content, as organic acids play dual positive roles in cell reprogramming. First, organic acids are inhibitors of the Krebs cycle, and this inhibition positively favors cell reprogramming. Organic acids are also exported from the cells into the culture medium, leading to a partial mimicking of a conditioned medium. A high protoplast plating density is also a critical factor for successful cell reprogramming. The optimal initial density for Arabidopsis protoplasts is 50000–100000 cells per mL. With this density, up to 40% of the cells undergo cell division. 

#### 2.1.7. Cell Viability/Activity Assays 

Retention of membrane integrity (cell viability) is critical for successful cultivation and indicates the culture medium suitability. Rapid loss of cell viability during the first 24 h of culture indicates problems with the culture medium (since only viable cells were selected by sucrose gradient treatment). Viability loss results in the release of dead cell contents into the medium, thereby enhancing the concentrations of nucleases, proteases, etc. that can harm viable competent cells.

**Figure 1:**
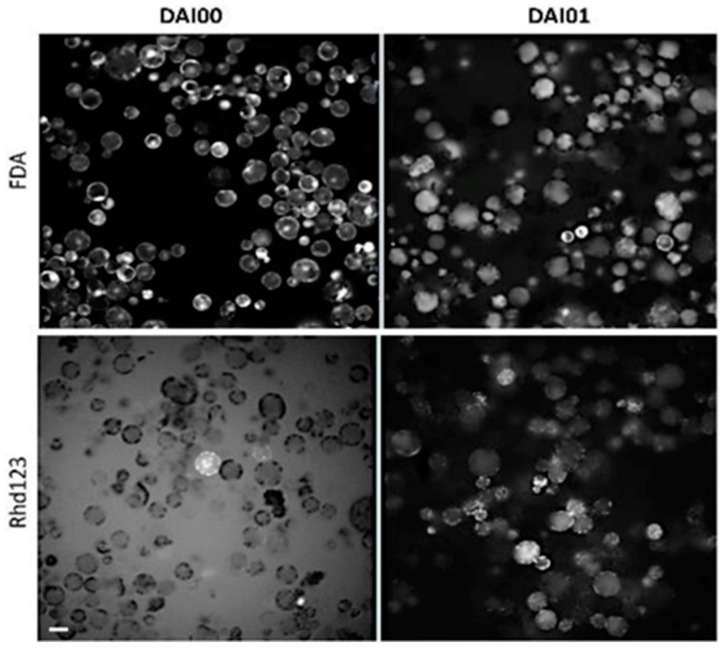
Protoplasts staining with FDA and rhd123. Plasma membrane stability and cell activity detected with fluorescein diacetate (FDA) and Rhodamine 123 (rhd123), respectively. 1 µM FDA and 1 µM rhd123 were added to the protoplast suspension at 1 h after isolation. Images were captured at 3 and 24 h after isolation. DAI- Day after isolation. The rhd123 image has a high background on DAI 0 because the dye was not taken up by the cells and remained in the medium. Scale bar—20 µm.

We used two strategies to monitor the kinetics of cell viability: labeling with fluorescein diacetate (FDA) dye that indicates leakiness from the cells and labeling with rhodamine 123 (rhd123) dye that characterizes cell activity (Figure 1). Fluorescein diacetate (FDA) is a non-fluorescent hydrophobic fluorescein derivative that readily passes through the cell membrane. In the cytoplasm, FDA is converted by intracellular esterases/proteases into fluorescein, which emits green fluorescence. Fluorescein is not retained in the cytoplasm but moves into different cell compartments; it is particularly accumulated in the chloroplast thylakoids, mitochondria, or vacuoles. For this reason, labeling is non-uniform and unsuitable for automatic analysis. The high negative charge of FDA fluorescein also interferes with cell activity and prevents cell proliferation. Therefore, the use of FDA is restricted to checking the quality of the isolated protoplasts and the suitability of the culture conditions during the first 24 h after isolation. 

In our preparations, almost 100% of the cells emitted a very bright FDA signal, mainly in the chloroplasts and cytoplasm. The presence of a bright FDA signal in almost 100% cells is explained by the selection of essentially 100% viable cells by the sucrose gradient step and by the maintenance of an optimal osmotic pressure for all cell types. After a 24 h incubation, the FDA had relocated to the vacuole in most cells. The dye was retained inside the protoplasts because they retained their membrane integrity; however, the high negative charge of FDA suppressed proliferation.

The use of rhd123 for accurate tracking and detection of cell functionality has advantages over the FDA because rhd123 dye does not prevent cell proliferation and it characterizes cell activity [32]. The rhd123 dye has been successfully used for cell tracking in animal cells [33]. In our hands, labeling with rhd123 did not prevent cell division at DAI 3, and we observed abundant cell division after 4–5 days in culture, with excellent visualization of the division plate.

**Figure 2:**
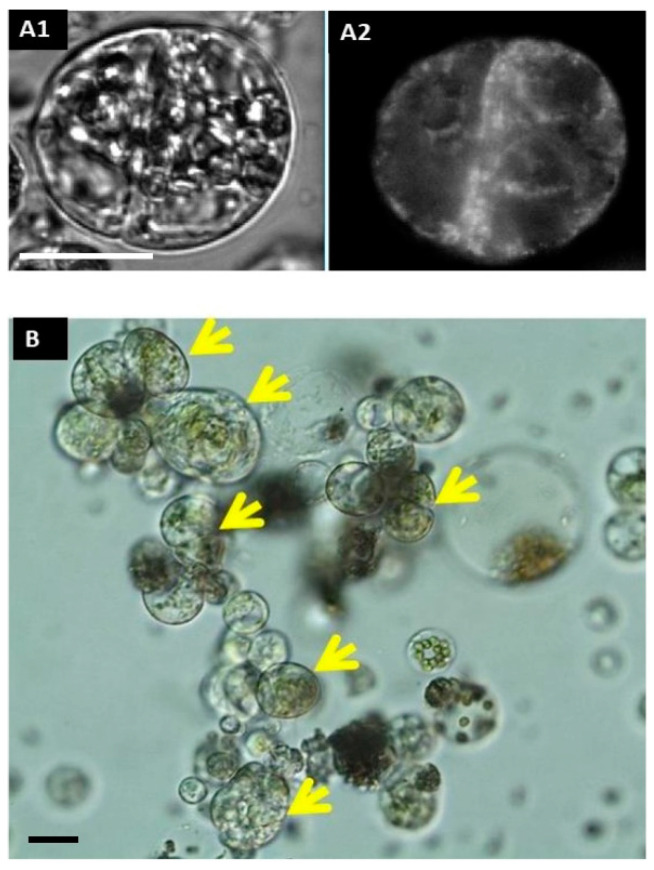
Cell division and microcolony formation. Tracking of the first cell division with rhd123 After a 2 h incubation: (**A1**) DIC (differential interference contrast); (**A2**) rhd123; (**B**) microcolony formation at 96 h after isolation. Scale bar: 20 µm. Yellow arrows mark compact colony.

In freshly isolated protoplasts (2–3 h after isolation), rhd123 shows a bright signal in only a few cells, with localization at the cell membrane. The absence of signals in most protoplasts is because the cells transiently lose their activity after isolation, and rhd123 reveals cell activities/membrane potentials. However, after a 24 h incubation, a bright signal was detected in most cells. The increase in the signal intensity suggests the activation of membrane-related transport and general cell activity. However, the most significant advantage of rhd123 is that it can be used to track and quantify cell proliferation (mitosis). After the next 2–3 days in the culture, cytokinesis was visible, with clearly labeled mitotic plates evident by rhd123 staining (Figure 2).

The best solvent for FDA and rhd123 is DMSO, for the following reasons: the solution is sterile, relatively stable (we have successfully stored it at –20°C for up to 1 year), and non-toxic to cells when the concentration of DMSO is less than 0.1%. In many published protocols, the authors have used polar solvents, like acetone or ethanol. However, the use of polar solvents might not be optimal for the following reasons: 1. Both solvents (and especially acetone) evaporate extremely rapidly, even in closed Eppendorf tubes and during pipetting, making the accurate addition of a small volume almost impossible. 2. Both solvents have adverse effects on cell membranes, especially in fragile systems like protoplasts, even at low concentrations.

#### 2.1.8. Analysis of Gene Expression during Cell Re-Programming

**Figure 3:**
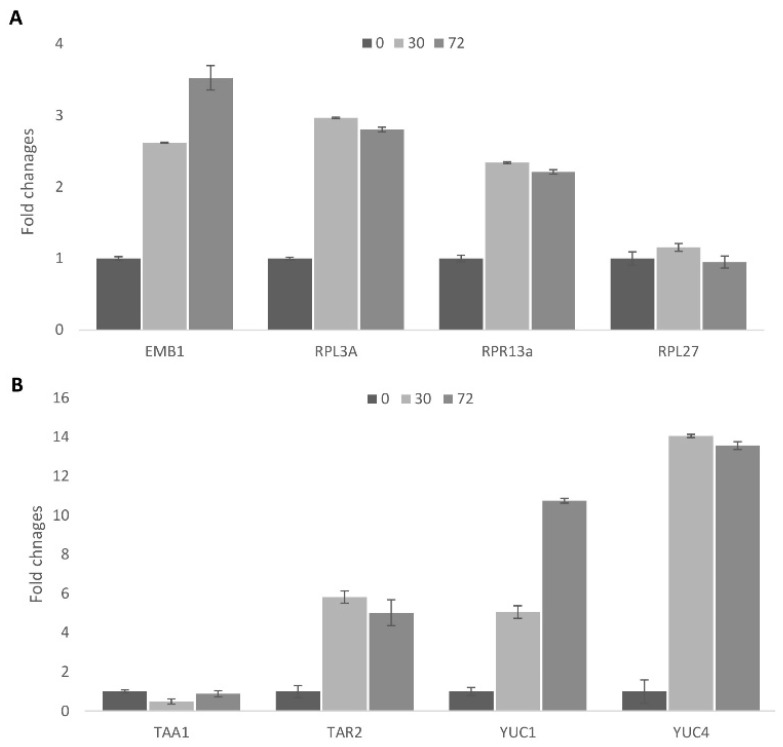
Analysis of gene expressions by qPCR in Arabidopsis protoplasts. The Actin (ACT2) gene, which does not change its expression in protoplasts, was used as a reference. X-axis: genes; Y-axis: fold changes; 0, 30 and 72 hours of incubation. Error bar means standard error. (**A**) nucleus related genes; (**B**) auxin biosynthesis genes.

Cell reprogramming is accompanied by significant changes in nuclear organization, the formation of nucleoli, and activation of ribosomal biogenesis [34,35]. In the present investigation, we chose four genes for detailed analysis of the processes related to general transcription activation: RPL3A (At1g43170), RPR13A (At4g00100.1), EMB1 (At2G18020.1), and RPL27A (At3g61110) (Figure 3). RPL3 and RPL13 are two of the most important, as they are involved in the regulation of nucleolar function, which has been confirmed as a requirement for cell proliferation in tobacco [36] and in rice [37]. 

Mesophyll protoplast re-programming includes two steps [2]. The first step involves a significant reprogramming of the nucleus, including chromatin relaxation [38], and is accompanied by the upregulation of enzymes related to nuclear architecture (e.g., RPL3a, RPL13a). Nuclear activation is related to cell preparation for cell division, as confirmed by DNA replication events. 

The second stage, as we have observed, involves the formation of highly compact cells similar to the *in planta* stem cells that is related to the induction of somatic embryogenesis. The qPCR results demonstrated an increase in the mRNA levels of cytoplasmic ribosomal protein genes during the first step of cell cultivation (0–30 h) (Figure 3A). At the same time, the mRNA levels of the plastid ribosomal gene remain unchanged. Interestingly, the mRNA levels of the EMB1 gene continue to increase even after the first cell division, and this may relate to the transition to stem-like cells. 

#### 2.1.9. Activation of Auxin Biosynthesis during Cell Activation 

Auxin is the main plant growth regulator responsible for cell cycle induction and maintenance. Exogenous auxin is necessary for plant regeneration, but so is endogenous auxin biosynthesis [39]. In the developing leaf, auxin biosynthesis accompanies cell cycle activities [40]. Among auxin biosynthesis genes, YUCCA 1,2, and 4 and TAR2 play a key role in auxin biosynthesis in leaves [41,42]. We demonstrated that these genes were also activated during cell cycle reactivation of the protoplasts from a differentiated state to cell cycle active status (Figure 3B).

Interestingly, the TAA1 gene did not show changes in expression during the early stage of activation, implying a redundant role of TAA1 in auxin biosynthesis. Internal auxin biosynthesis through the YUCCA family and TAR2 was active in dividing leaf cells, so these genes were also expected to be activated during cell conversion from the differentiated to the dividing status. An auxin response gene (IAA2), as well as an auxin receptor gene (TIR1), were also strongly upregulated [43]. Upregulation of TIR1 indicates the possible occurrence of auxin signaling due to an increased auxin concentration in the protoplasts as well as to an increased sensitivity to auxin. Overall, simultaneous changes in the expression of the genes responsible for nuclei organization and auxin biosynthesis suggested the close link between auxin and epigenetic regulation on the single cell level [44]. 

#### 2.1.10. Detection of Protoplast Competence to Undergo the Cell Cycle

**Figure 4:**
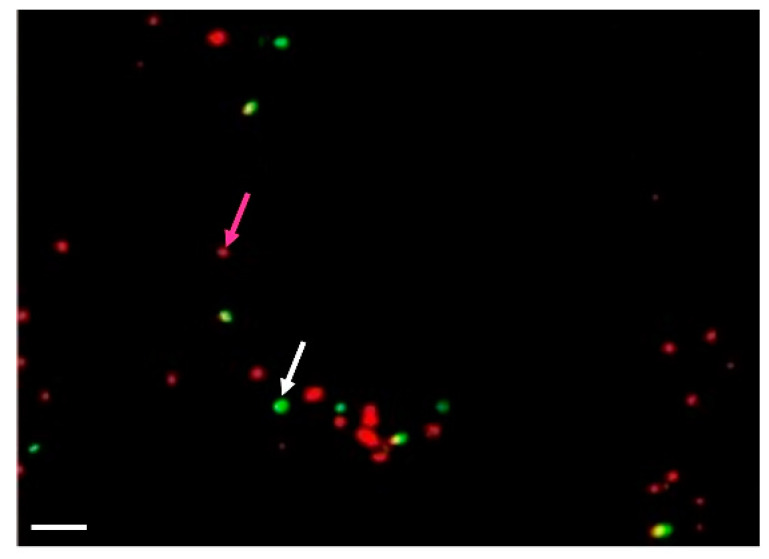
Detection of the cell competence to undergo the cell cycle progression in Arabidopsis shoot-derived protoplasts. 5-Ethynyl-2´-deoxyuridine (EdU) was added to the cell suspension at 12 h after isolation. The cells were fixed at 36 and 60 h after isolation, and the nuclei were isolated and subjected to the standard procedure for EdU detection. Nuclei are red; EdU stained nuclei are in green. White arrow marked positive nuclei, while magenta arrow— negative. Scale bar—20 µm.

Activation of membrane activity (as detected by rhd123 fluorescence) and upregulation of cytoplasmic ribosomal proteins indicate that the protoplasts achieved competence for cell cycle progression. In diploid cells (G1), the first step of the cell cycle is DNA replication. We used a time course of (5-ethynyl-2’-deoxyuridine) (EdU) incorporation to detect the kinetics of DNA replication and detected the first EdU-incorporation at as early as 36 h of incubation (up to 14%). The incorporation ratio was increased to up to 50% after 60–72 h of incubation (Figure 4).

#### 2.1.11. Nutrient Adjustment and Induction of Cell Division

An optimal hormonal balance, in combination with optimal macro/micronutrient adjustments, is crucial for protoplasts to reenter the cell cycle. Auxin is a key signal for cell reprogramming, as no auxin-independent cell division was detected. We found that 2 µM 2,4-D was the optimal concentration in our medium and that concentrations of 5 µM had a visible toxic effect. We used 0.7 µM BAP as our cytokinin source. Several 2,4-D concentrations have been tested. We found that under 10 µM 2-4,D cell proliferation cannot be detected, and cells rapidly undergo browning. The maximal yield of proliferation was detected at 2 µM 2,4-D in combination with TK30 medium and it reached 40.6 ± 1.7% after 96 hours in the culture. 

However, further maintenance of the cell on this medium led to slowing down of cell division with no colony formation. Therefore, we removed 2,4-D and transferred the cells to a cytokinin-containing medium. This transfer allowed rapid growth of a colony, which reached a size of approximately 0.5 mm in the following week. 

However, the auxinic compound 2,4-D has a significant inhibitory effect on cell division in dividing cells. Therefore, continuous cultivation of cells on a medium with high auxin content at high density led to micro-colony browning and a loss of viability. We overcame this challenge by removing the cells from the original medium at DAI 5, washing, and then embedding the cells in alginate at a density (3000 cells/ml) at least 30 times less than the initial density. We also removed the 2,4-D and used 0.5 µM NAA in combination with 2 µM BAP, as well as transferring the cells to light. 

#### 2.1.12. Type of Cells and Plant Regeneration 

We could distinguish two types of cells: vacuolated cells with large vacuoles and compact cells with numerous small vacuoles [26]. Our optimized conditions ensured the selection of the compact cell type, as these cells underwent cell division and formed compact colonies that favored further plant regeneration. This type of cell has a close similarity to the stem cells observed *in planta* and potentially can be totipotent. (Figure S2). 

*In planta,* stem-like cells are able to build new organs and serve as auxin sources. To prove that cells after cultivation in TK30 medium become relatively auxin-independent (start internal auxin synthesis, see Figure 3B), we washed them and embedded them in alginate after compact cell division. 17.2% ± 1.2% of the cells from the TK30 medium, which mainly showed compact cell division, were able to grow on TK4 medium supplemented with cytokinin and form compact dense micro calli. At the same time, only few microcalli were detected after the non-compact cell division. 

We ensured rapid shoot formation and avoided cell adaptation to external auxin (which may prevent shoot induction) by washing the cells after 4–5 days of culture to remove external auxin, diluting the cell density at least tenfold, and embedding the diluted cells in alginate. The TK30 medium was replaced with TK4 medium contain cytokinin (as a potential inducer of auxin synthesis). The combinations of macro and micronutrients provided in the TK4 medium (Table 3) are sufficient for compact colony growth as well as further shoot induction. The microcolony cultivation on MS medium led to the formation of rather unorganized soft callus, as described by Murashige and Skoog [15]. 

**Figure 5:**
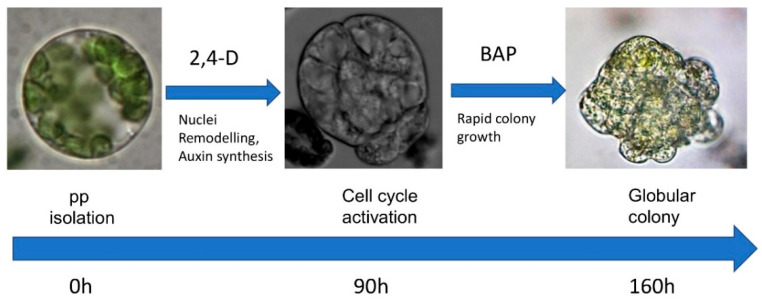
Pipeline for Arabidopsis shoot-derived protoplast cultivation.

The pipeline for the cultivation of Arabidopsis shoot-derived protoplasts for plant regeneration is presented in Figure 5. 

### 2.2. Application of Shoot-Derived Protoplasts to Investigate the Effect of Different Compounds on Cell Reprogramming 

We further demonstrated the usefulness of this protocol as a way to investigate different factors that are potentially involved in cell reprogramming by testing the effects of GSH on plant regeneration from shoot-derived protoplasts. We recently demonstrated the involvement of GSH in the regulation of auxin response in the roots of Arabidopsis seedlings [46]. Here, we dissected the mechanism of GSH action at the cellular level by investigating the effect of exogenous GSH on the re-entry of Arabidopsis mesophyll protoplasts into the cell cycle. We choose leaves as the starting material to ensure inclusion of all steps of cell de-differentiation as well as generation of quantitative data on cell proliferation. Evaluation of the GSH levels during cell cycle activation showed a significant rise in GSH during cultivation from 1.95 nmol/10^6^ cells in freshly isolated cells to 16.4 nmol/10^6^ cells after 72 h of culture, as measured by HPLC in our previous study [45]. We addressed the possibility that this was simply a correlation, rather than a direct GSH effect, by applying external GSH (1 mM) and monitoring the cell proliferation ratio. The exogenous GSH significantly increased the ratio of cells that passed through DNA replications from 28.7 in the untreated control to 47.1 in cells treated with GSH. The cell proliferation ratio was also higher after 84 h in culture, from 2.42% in the control cells to 13.1% in cells treated with 1 mM GSH (see Figure S3). Because auxin is the main regulator of cell cycle re-entry, we concluded that GSH can increase the sensitivity to auxin, as was observed previously in roots [46].

### 2.3. Root-Derived Protoplasts

Root protoplasts are an alternative to shoot protoplasts for some species. For example, root protoplasts can be easily isolated from Medicago sp. plants. By contrast, Arabidopsis is not very suitable for root protoplast isolation, as the root cell population is highly heterogeneous, and only a limited number of cells can be isolated from the elongation zone. This situation arises from quite different cell wall thicknesses in different root zones/cell types, which affects the “digestibility” by cellulolytic enzymes. 

**Figure 6:**
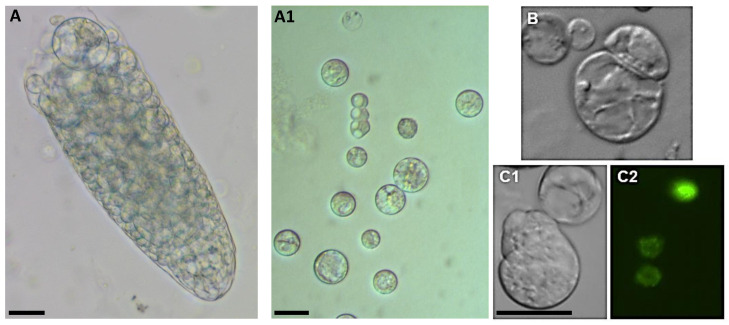
Cell proliferation from root-derived Arabidopsis protoplasts. (**A**)—in enzyme solution; (**A1**)—in the medium after isolation; (**B**)**—**first cell division; (**C1**,**C2**)—marker line H2B::YFP undergo mitosis. Scale bar— 20 µm.

In addition, the Arabidopsis root, unlike the roots of other species, has only a single cortical layer, so the root protoplast population is very heterogeneous. For this reason, only a small number of protoplasts remain competent for cell reprogramming after their isolation from roots. However, isolation is possible, and cell division can be achieved (Figure 6) by first inducing activated pericycle cells to formed callus-like structures and then isolating the protoplasts from the calluses. However, these protoplasts cannot be viewed as true root protoplasts as they are the protoplasts of pericycle-derived callus cells [8]. 

## 3. Materials and Methods 

### 3.1. Plant Material and Growth Conditions

The plants used in these experiments were *Arabidopsis thaliana* accession Col-0 (L.) Heyhn. and H2B::YFP [47] lines. Seeds were surface-sterilized and sown on solid Arabidopsis medium (TK1, 1% sucrose, 1 mM MES and 1.1% agar, pH 5.6, adjusted with KOH) (Table 1, [9]). After vernalization for 16 h at 4°C, the seeds were germinated on vertically oriented plates at 20–21°C under 16 h:8 h light/dark period with a light intensity of 80 μmol m^−2^ s^−1^. 108 media with different N:P:K ratio, different Cl supplementation, and different micronutrients were tested for optimal plant growth. All experiments were done in triplicate; analysis of the optimal medium for plant growth were presented in [9]. 

### 3.2. Protoplasts Isolation and Cultivation

#### 3.2.1. Enzyme Preparation

The enzyme solution for leaf protoplast isolation contained 0.5 M sucrose for osmotic adjustment, 0.5% Cellulase R10 (C8001, Duchefa Haarlem, the Netherland Company), 0.4% Macerozyme R10 (M8002, Duchefa Haarlem, the Netherland Company), 5 mM MES, and 5 mM Ca(NO3)2 pH 5,4. The modified W5 (W5m) solution [39] contained 125 mM Ca(NO_3_)_2_, 154 mM NaCl, 5 mM KCl, 5 mM glucose, and 2 mM MES. The enzyme solution for root protoplast isolation contained modified W5m salts as osmotic adjustment, 1 g/L sucrose, 0.9% Cellulase R10; 0.4% Macerozyme R10; 0.5% Pectinase (P4716, Sigma-Aldrich St. Louis, Mo., U.S.A), 0.6% Driselase (D9515, St. Louis, Mo., U.S.A), and 5 mM MES, pH 5.4. All enzyme solutions were pre-warmed at 55°C for 10 min to inactivate DNAse/proteases and then filtered through 0.22 µm filters. Sterile enzyme solutions could be stored at 4 °C for up to 1 month without any reduction in activity. 

#### 3.2.2. Protoplasts Isolation 

Shoots from 8–10-days-old seedlings (200–300 mg, one 120 mm square Petri dish contains appr 100 seedlings) grown on TK1 medium were transferred to a 40 mm Petri dish containing 0.8 ml of cutting buffer (0.3 M glucose, 5 mM Ca(NO_3_)_2_, pH 5.4). The explants were gently cut into small pieces (strips) (approximately 1 mm width), incubated for 15 minutes, gently washed twice with the same buffer, and then the buffer was replaced with enzyme solution. The washing step removed DNAse/proteases arising from damaged cells. The dishes were incubated in the dark at 23°C for 9–12 hours, without shaking. To improve the yield of competent cells, we gently removed the enzyme solution and added 0.45 M Glucose + 5 mM Ca(NO3)2 for 1 hour (optional). Thereafter, this solution was replacement by W5m solution, followed by gentle shaking. The resulting protoplast suspensions in W5m solution were layered on top of 0.5 M sucrose buffer and centrifuged at 20 g for 5 minutes.

Viable protoplasts accumulated in the interphase between the sucrose and W5m. The protoplast fraction was carefully collected and filtered through 67 µm filters homemade from nylon mesh (Millipore, Molsheim, France). The protoplast suspension was then subjected to two more washing steps by resuspension in 5–7 mL W5m solution and repeated centrifugation. The protoplasts were finally resuspended in TK30 culture medium (Table 2) with the appropriate hormone content at a cell density of 1 × 10^5^ cells per ml. 

Roots from 8–10-day-old seedlings were cut into pieces in W5mod solution in 40 mm Petri dishes, and then the W5mod was replaced with enzyme solution. The dishes were incubated in the dark at 25°C for 10–14 hours without shaking. Subsequently, the dishes were shaken and the resulting root protoplast suspension was filtered through 67 µm filters and washed twice with W5m by repeated centrifugations. Using the W5m retained the integrity of almost all cells, so the purification step could be omitted. The root protoplasts were resuspended in TK30 medium supplemented with appropriate concentrations of hormones. 

All experiments were performed in three biological replicates, including testing the different procedure of protoplast isolation with at least 10^6^ cells in each. 

#### 3.2.3. Protoplasts Cultivation

The shoot and root protoplasts were cultured in 40 mm Petri dishes containing 2 ml TK30 medium supplemented with 2 µM 2,4-D and 0.7 µM BAP under dim light at 21–22°C for 3–4 days. For the DNA replication assay, small aliquots of the protoplast suspension (0.3 ml) were distributed in wells in a 24-well plate and cultured under similar conditions as the main suspension. Hormonal contents of the medium have been adjusted by testing different 2,4-D concentration (0.5; 2 and 10 µM), among which 2 µM was found to be optimal. 

For the osmotic adjustment, we tested TK30 medium with 0.4 M glucose, 0.4 M glucose + 0.1 M mannitol or 0.4 M glucose + 0.1 M mannitol + 0.05 M sucrose. 

For the Glutathione treatment, stock solution of the GSH (50 mM) was prepared by dissolving it in 5 mM MES-buffer, pH was adjusted to 5.6, and the solution was filter sterilized and added to the medium in appropriate concentration. All experiments were performed at least in triplicate. 

#### 3.2.4. “Creation” of Totipotent Cells 

After the first cell division (approximately 3–4 days after protoplast isolation), the cells were washed with a new TK30 medium and embedded in alginate beds. Briefly, cells were collected into Falcon tubes, centrifuged (10 g, 3 min), and re-suspended in a new TK30 medium at a cell density of 4 × 10^3^–1 × 10^4^ cells per ml. An equal volume of 1.5% alginate solution containing 0.4 M mannitol was added and gently mixed. The resulting protoplast suspension was dispersed on the surface of 1% agarose containing 0.4 M mannitol + 40 mM CaCl_2_ in a 40 mm Petri plate.

After solidification (approximately 30 min), the alginate beds were transferred to new plates containing liquid medium supplemented with 2.5 µM BAP and 0.5 µM NAA and transferred to light (100 μmol m^−2^ s^−1^, 16 h/8 day/night cycle, 23°C). 

### 3.3. Cell Viability/activity Determination

Fluorescein diacetate (FDA) (Thermo-Fischer Scientific, F1303, Thermo Fisher Scientific Inc., Waltham, MA, USA) and Rhodamine 123 (R8004, Sigma-Aldrich, St. Louis, Mo., U.S.A) were used to determine cell viability/activity. Both dyes were dissolved in DMSO at 2 mM as a stock solution, then diluted in 50 µl of TK30 medium, and added to the culture at a final concentration of 1 µM. Plates were inspected after 1 hour, 24 hours, and 72 hours (for rhd123) with a ZEISS Axiovert 200 microscope equipped with a specific GFP filter. 

### 3.4. Cell Proliferation Assay

The kinetics of DNA replication has been used to determine the competence of cells to undergo cell cycle progression. In the present study, 5µM EdU was added to the protoplast culture after a 6 h cultivation. The cells were collected at the desired time points, transferred to Eppendorf tubes, centrifuged at 100 g for 5 min, and then the medium was replaced with MTSB buffer containing 4% formaldehyde and 0.4% Triton X-100 [48]. After a 20 min fixation/permeabilization period, the tubes were centrifuged for 5 min at 100 g and then washed twice with H_2_O by centrifugation. The cells were then incubated for 30 min in EdU-detection solution (C10337, Thermo Fisher Scientific Inc., Waltham, MA, USA) and washed once with H_2_O by centrifugation. The cells were then re-suspended in 0.1 mg/l DAPI in H_2_O, washed with H_2_O, and resuspended in DAPI-GOLD mounting medium. Finally, the cells were loaded onto microscopic slides, covered with coverslips, and inspected by a ZEISS Axiovert 200 microscope equipped with a specific GFP filter. All labelling was done in triplicates. 

Calculations of cells proliferation ratio (cells undergo mitosis) have been done by microscopic inspection of at least 10 randomly selected areas and calculation of cell with visible mitotic plate in each. Similarly, calculation of DNA replication index was done as a ratio between EdU-positive nuclei and total amount on nuclei (DAPI) in 8–10 randomly selected areas.

### 3.5. Quantitative Real-Time PCR Assay (qPCR)

Protoplasts were collected at the indicated time points (0, 30 and 72 hours after isolation) by centrifugation. TRIzol reagent (Invitrogen) was used to isolate total RNA. First-strand cDNA was synthesized with oligo(dT)18 primers using the First Strand cDNA Synthesis Kit (Fermentas, Waltham, MA, USA). Quantitative real-time PCR was carried out using the POWER SYBR GREEN PCR master mix (Roche, Basel, Switzerland) and gene-specific primers (Table 4) in a Light Cycler 480 Real-Time PCR System (Roche, Basel, Switzerland). Gene expression data were analyzed statistically according to Livak et al. (2001) [49] based on the actin2 (ACT2) gene, which is the most stable gene in Arabidopsis protoplasts [50]. Experiments were done in two independent biological replications with three technical replications in each. 

## 4. Conclusions

We described a robust protocol for the isolation and cultivation of protoplasts from the model plant *Arabidopsis thaliana* using either shoots or roots as starting material. We optimized the culture media that support donor plant growth, and all cell culture steps, and our finding emphasized the importance of identifying optimal conditions for successful plant cultivation, protoplast isolation, initiation of cell division, and other reprogramming processes. Investigation of gene expression and cell cycle kinetics suggests that the three steps associated with cell conversion from differentiation to proliferation should be optimized separately, taking into account the underpinning mechanisms of cell reprogramming. 

## Figures and Tables

**Table 1:** Composition of different nutrients media (in mg/L) for in vitro growth of Arabidopsis seedlings (TK). Arabidopsis medium (AM; half-strength Murashige and Skoog Medium) [15] and for the optimized medium described in [9] (TK1).

Compound	AM (½ MS)	TK1
Macronutrients (mg/L)		
NH_4_NO_3_	825	-
KNO_3_	950	900
NH_4_H_2_PO_4_	-	230
MgSO_4_·7H_2_O	180	200
KH_2_PO_4_	68	-
Ca(NO_3_)_2_ 4H_2_O	-	250
CaCl_2_ 2H_2_O	220	-
Fe-Chelate (*)	2.5 ml	2 ml
Micronutrients	½ micro MS	½ micro TK
Organic compounds (mg/L)		
Bacto-tryptone	-	150
Vitamines (**)	½ B5 vitamines	½ B5 vitamines
Myoinositol	50	50
Sucrose	10000	10000
MES	200	200
pH	5.6	5.6
(a). Micronutrient concentrations in the medium (***).		
Micronutrients (mg/L)	micro MS	micro TK
H_3_BO_3_	6.2	4
MnCl_2_ 4H_2_O	16.8	18
ZnSO_4_ 7H_2_O	30	1.8
Na_2_MoO_4_ 2H_2_O	0.25	0.25
NaI	0.76	0.8
CuSO_4_ 5H_2_O	0.025	0.2
CoCl_2_ 6H_2_O	0.025	0.2

* Fe-chelate—20 mM stock solution. ** B5 vitamins: 10 mg/L B1; 1 mg/L- B6 and 1 mg/l PP (prepared as 1000 × stock solution). *** Micronutrients stock has been prepared separately (prepared as 1000 × stock solution).

**Table 2:** TK30 medium for protoplasts cultivation.

Components	Concentrations (mg/L)
NH_4_H_2_PO_4_	200
Ca(NO_3_)_2_·4H_2_O	400
KNO_3_	1500
MgSO_4_·7H_2_O	300
KH_2_PO_4_	200
CaCl_2_·2H_2_O	80
Fe-chelat (*)	4 mL from 20 mM stock *
Glucose	72000
Mannitol	18000
Sucrose	12000
Micronutrients	½ micro TK1 **
Glutamine	100
Vitamins	1 mL from the stock ***
Myoinositol	200
Bacto-tryptone	150
Na-citrate	40
Na-pyruvate	20
Na-fumarate	40

* 20 mM stock solution as in Table 1. ** from × 1000 stock, see Table 1(a); *** from × 1000 stock, see Table 1.

**Table 3:** TK4 medium for plant regeneration for 1 L of medium.

NH_4_ H_2_PO_4_	250 mg
(NH_4_ )_2_ SO_4_	400 mg
KNO3	2000 mg
MgSO_4_·7H_2_O	450 mg
Ca(NO_3_)_2_ ·4H_2_O	350 mg
Fe-chelat	3 ml *
Micro TK (see Table 1(a))	1 ml **
MES	200 mg
B5 Vitamins	1ml ***
Bacto tryptone	200 mg
Myoinositol	100 mg
Sucrose	30 g
pH	5.7

*—from 20 mM stock. **—from × 1000 stock, see Table 1(a). ***—from × 1000 stock, see Table 1.

**Table 4:** Primers for qPCR.

Gene	Forward	Reverse
IAA2	CGACGCTCCTGCTCTAGACT	AAAACCCCGAAGTTTCGTCT
TIR1	AGATAAGGGACTGCCCGTTT	GACCAGCCACTGTTCGGTAT
ACT2	CGCTATGTATGTCGCCA	CTTGCCCATCGGGTAA
EMB1	AACGAGCTCTTCGTCGTCGCCGC	GAGAGGACACCACGATCACC
YUC1	CCTAGAACGGTCGGATTCAA	AGTGGGAAGCGTAGGACTCA
YUC2	TGCTCAAGTGGTTTCCAGTG	CCAACGTCCAAAACAGGAGT
YUC4	TGGGCAATACCGACCTTTTA	AAAGAAATGGCACCGACATC
TAA1	GATGAAGAATCGGTGGGAGAAGC	CGTCCCTAGCCACGCAAACGCAGG
TAR2	CATGATTTGGCTTACTATTGGCCACAG	GTCTTTCACCAAAGCCCATCCAATC
RPL27	ACCCACCTGCTGAGCTTGAGA	GGCAGTTTCCGCACACCACA
RPL3A	TTGGTGCGTGGCATCCTGCT	TGGCTGTGTGTGCCTCAGTACCA
RPS13A	GCTCATGGCCTTGCTCCTGAGA	GCGAGCGAGGCGGTGAATCC

Legends: ACT2 (Actin2) (At3g18780); YUC1—auxin biosynthesis gene—At4g32540; YUC 2 auxin biosynthesis gene—At4g13260; YUC4 auxin biosynthesis gene—At5g11320; TAR2 TRYPTOPHAN AMINOTRANSFERASE RELATED 2—(At4G24670; TAA1—TRYPTOPHAN AMINOTRANSFERASE OF ARABIDOPSIS 1—At1g70560; IAA2—INDOLE-3-ACETIC ACID INDUCIBLE 2—At3g23030; TIR1- Transport Inhibitor Response 1—At3g62980; RPL3A—RIBOSOMAL PROTEIN 1—At1g43170; RPR13A—RIBOSOMAL PROTEIN S13A—At4g00100.1; EMB1 EMBRYO DEFECTIVE 2296-At2G18020.1; RPL27A—RIBOSOMAL PROTEIN S27—At3g61110.

## Data Availability

Not applicable.

## Supplementary Materials

The following are available online at https://www.mdpi.com/2223-7747/10/2/375/s1, Figure S1. Cell proliferation ratio after 120 hours in the culture. Figure S2. Example of two types of cell division: A- vacuolated; B- compact with de-differentiated chloroplasts. Scale bar- 20 μm Figure S3. Effect of the GSH on DNA replication and cell proliferation. Cells were cultured on the TK30 medium supplemented with 2 μM 2,4-D or 2 μM 2,4-D + 1 mM GSH. DNA replication and cell proliferation ratio were counted after 84 hours of cultivation. % of positive cells means % of cell pass DNA replication or mitosis. (N = 4). Error bar means standard error.

## References

[1] Birnbaum K.D., Roudier F. (2017). Epigenetic memory and cell fate reprogramming in plants. Regeneration.

[2] Pasternak T., Lystvan K., Betekhtin A., Hasterok R. (2020). From Single Cell to Plants: Mesophyll Protoplasts as a Versatile System for Investigating Plant Cell Reprogramming. Int. J. Mol. Sci..

[3] Negrutiu I., Beeftink F., Jacobs M. (1975). *Arabidopsis thaliana* as a model system in somatic cell genetics I. Cell and tissue culture. Plant Sci. Lett..

[4] Damm B., Willmitzer L. (1988). Regeneration of fertile plants from protoplasts of different *Arabidopsis thaliana* genotypes. Mol. Gen. Genet..

[5] Karesch H., Bilang R., Potrykus I. (1991). *Arabidopsis thaliana*: Protocol for plant regeneration from protoplasts. Plant Cell Rep..

[6] O′Neill C.M., Mathias R.J. (1993). Regeneration of plants from protoplasts of *Arabidopsis thaliana* L. cv. Columbia (C24), via direct embryogenesis. J. Exp. Bot..

[7] Chupeau M.C., Granier F., Pichon O., Renou J.P., Gaudin V., Chupeau Y. (2013). Characterization of the early events leading to totipotency in an Arabidopsis protoplast liquid culture by temporal transcript profiling. Plant Cell.

[8] Mathur J., Koncz C., Szabados L. (1995). A simple method for isolation, liquid culture, transformation and regeneration of *Arabidopsis thaliana* protoplasts. Plant Cell Rep..

[9] Pasternak T., Ruperti B., Palme K. (2020). A simple high efficiency and low cost in vitro growth system for phenotypic characterization and seed propagation of *Arabidopsis thaliana*. Biorxiv.

[10] Masson J., Paszkowski J. (1992). The culture response of *Arabidopsis thaliana* protoplasts is determined by the growth conditions of donor plants. Plant J..

[11] Donnelly P.M., Bonetta D., Tsukaya H., Dengler R.E., Dengler N.G. (1999). Cell cycling and cell enlargement in developing leaves of Arabidopsis. Dev. Biol..

[12] Moreno S., Canales J., Hong L., Robinson D., Roeder A.H., Gutiérrez R.A. (2020). Nitrate Defines Shoot Size through Compensatory Roles for Endoreplication and Cell Division in *Arabidopsis thaliana*. Curr. Biol..

[13] Hewitt E.J. (1963). The essential nutrient elements: Requirements and interactions in plants. Plant Physiology.

[14] Hewitt E.J. (1966). Sand and Water Culture Methods Used in the Study of Plant Nutrition.

[15] Murashige T., Skoog F. (1962). A revised medium for rapid growth and bioassays with tobacco tissue cultures. Physiol. Plant..

[16] Dalton C.C., Iqbal K., Turner D.A. (1983). Iron phosphate precipitation in Murashige and Skoog media. Physiol. Plant..

[17] Raizada M.N., Goron T.L., Bannerjee O., Mason M.Q., Pautler M., Brazolot J., Morris A.D., Kajenthira A., Dinka S.J., DiMeo N. (2017). Loss of developmental pluripotency occurs in two stages during leaf aging in *Arabidopsis thaliana*. Vitr. Cell. Dev. Biol. Plant.

[18] Del Pozo J.C., Diaz-Trivino S., Cisneros N., Gutierrez C. (2006). The balance between cell division and endoreplication depends on E2FC-DPB, transcription factors regulated by the ubiquitin-SCFSKP2A pathway in Arabidopsis. Plant Cell.

[19] Zhao J., Morozova N., Williams L., Libs L., Avivi Y., Grafi G. (2001). Two phases of chromatin decondensation during dedifferentiation of plant cells distinction between competence for cell fate switch and a commitment for S phase. J. Biol. Chem..

[20] Yoo S.D., Cho Y.H., Sheen J. (2007). Arabidopsis mesophyll protoplasts: A versatile cell system for transient gene expression analysis. Nat. Protoc..

[21] Menczel L., Nagy F., Kiss Z.R., Maliga P. (1981). Streptomycin resistant and sensitive somatic hybrids of *Nicotiana tabacum*+ *Nicotiana knightiana*: Correlation of resistance to N. tabacum plastids. Theor. Appl. Genet..

[22] Kao K.N., Michayluk M.R. (1975). Nutritional requirements for growth of *Vicia hajastana* cells and protoplasts at a very low population density in liquid media. Planta.

[23] Luo Y., Koop H.U. (1997). Somatic embryogenesis in cultured immature zygotic embryos and leaf protoplasts of *Arabidopsis thaliana* ecotypes. Planta.

[24] Franco-Navarro J.D., Brumos J., Rosales M.A., Cubero-Font P., Talón M., Colmenero-Flores J.M. (2016). Chloride regulates leaf cell size and water relations in tobacco plants. J. Exp. Bot..

[25] Kaiser S., Scheuring D. (2020). To Lead or to Follow: Contribution of the Plant Vacuole to Cell Growth. Front. Plant Sci..

[26] Pasternak T.P., Prinsen E., Ayaydin F., Miskolczi P., Potters G., Asard H., Van Onckelen H.A., Dudits D., Fehér A. (2002). The role of auxin, pH, and stress in the activation of embryogenic cell division in leaf protoplast-derived cells of alfalfa. Plant Physiol..

[27] Andreini C., Bertini I., Rosato A. (2009). Metalloproteomes: A bioinformatic approach. Acc. Chem. Res..

[28] Stormea N.D., Masonb A. (2014). Plant speciation through chromosome instability and ploidy change: Cellular mechanisms, molecular factors and evolutionary relevance. Curr. Plant Biol..

[29] Rose R.J. (2008). *Medicago truncatula* as a model for understanding plant interactions with other organisms, plant development and stress biology: Past, present and future. Funct. Plant Biol..

[30] Sheen J. (2001). Signal transduction in maize and Arabidopsis mesophyll protoplasts. Plant Physiol..

[31] Kondratenko S.I., Pasternak T.P., Samovol O.P., Mogilna O.M., Sergienko O.V. (2020). Modeling of asymmetric division of somatic cell in protoplasts culture of higher plants. Regul. Mech. Biosyst..

[32] Ferlini C., Biselli R., Nisini R., Fattorossi A. (1995). Rhodamine 123: A useful probe for monitoring T cell activation. Cytom. J. Int. Soc. Anal. Cytol..

[33] Cai J., Limke T.L., Ginis I., Rao M.S. (2003). Identifying and tracking neural stem cells. Blood Cells Mol. Dis..

[34] Pasternak T., Miskolczi P., Ayaydin F., Mészáros T., Dudits D., Fehér A. (2000). Exogenous auxin and cytokinin dependent activation of CDKs and cell division in leaf protoplast-derived cells of alfalfa. Plant Growth Regul..

[35] Pasternak T., Asard H., Potters G., Jansen M.A. (2014). The thiol compounds glutathione and homoglutathione differentially affect cell development in alfalfa (*Medicago sativa* L.). Plant Physiol. Biochem..

[36] Popescu S.C., Tumer N.E. (2004). Silencing of ribosomal protein L3 genes in *N. tabacum* reveals coordinate expression and significant alterations in plant growth, development and ribosome biogenesis. Plant J..

[37] Zheng M., Wang Y., Liu X., Sun J., Wang Y., Xu Y., Lv J., Long W., Zhu X., Guo X. (2016). The RICE MINUTE-LIKE1 (RML1) gene, encoding a ribosomal large subunit protein L3B, regulates leaf morphology and plant architecture in rice. J. Exp. Bot..

[38] Pasternak T., Dudits D. (2019). Epigenetic Clues to Better Understanding of the Asexual Embryogenesis *in planta* and in vitro. Front. Plant Sci..

[39] Wójcikowska B., Wójcik A.M., Gaj M.D. (2020). Epigenetic Regulation of Auxin-Induced Somatic Embryogenesis in Plants. Int. J. Mol. Sci..

[40] Ljung K., Bhalerao R.P., Sandberg G. (2001). Sites and homeostatic control of auxin biosynthesis in Arabidopsis during vegetative growth. Plant J..

[41] Cheng Y., Dai X., Zhao Y. (2007). Auxin synthesized by the YUCCA flavin monooxygenases is essential for embryogenesis and leaf formation in Arabidopsis. Plant Cell.

[42] Mashiguchi K., Tanaka K., Sakai T., Sugawara S., Kawaide H., Natsume M., Hanada A., Yaeno T., Shirasu K., Yao H. (2011). The main auxin biosynthesis pathway in Arabidopsis. Proc. Natl. Acad. Sci. USA.

[43] Cao X., Yang H., Shang C., Ma S., Liu L., Cheng J. (2019). The Roles of Auxin Biosynthesis YUCCA Gene Family in Plants. Int. J. Mol. Sci..

[44] Mateo-Bonmatí E., Casanova-Sáez R., Ljung K. (2019). Epigenetic regulation of auxin homeostasis. Biomolecules.

[45] Pasternak T., Potters G., Caubergs R., Jansen M.A. (2005). Complementary interactions between oxidative stress and auxins control plant growth responses at plant, organ, and cellular level. J. Exp. Bot..

[46] Pasternak T., Palme K., Paponov I.A. (2020). Glutathione Enhances Auxin Sensitivity in Arabidopsis Roots. Biomolecules.

[47] Boisnard-Lorig C., Colon-Carmona A., Bauch M., Hodge S., Doerner P., Bancharel E., Dumas C., Haseloff J., Berger F. (2001). Dynamic analyses of the expression of the HISTONE: YFP fusion protein in Arabidopsis show that syncytial endosperm is divided in mitotic domains. Plant Cell.

[48] Pasternak T., Tietz O., Rapp K., Begheldo M., Nitschke R., Ruperti B., Palme K. (2015). Protocol: An improved and universal procedure for whole-mount immunolocalization in plants. Plant Methods.

[49] Livak K., Schmittgen T.D. (2001). Analysis of relative gene expression data using Real-Time Quantitative PCR and the 2—ΔΔCT method. Methods.

[50] Sheahan M.B., Collings D.A., Rose R.J. (2020). ACTIN7 Is Required for Perinuclear Clustering of Chloroplasts during Arabidopsis Protoplast Culture. Plants.

