# Supplementary material for: Optimizing Protocols for Arabidopsis Shoot and Root Protoplast Cultivation
Source: Plants (Basel). 2021 Feb 15;10(2):375. doi: 10.3390/plants10020375 (PMC7919498; doi:10.3390/plants10020375)
Supplement: Supplementary file 1 [file plants-10-00375-s001.pdf]

plants MDPI

Supplementary figures.

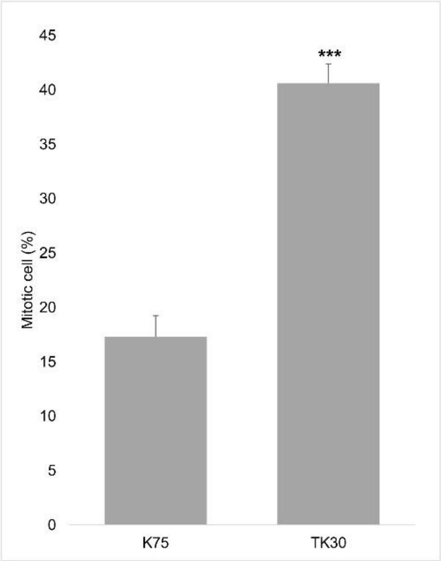

Figure S1. Cell proliferation ratio after 120 hours in the culture.

Cell proliferation ratios were monitored and calculated after 120 hours in the culture. The numerical data in the diagrams are presented as mean ± SE (n = 5). Error bar means standard error

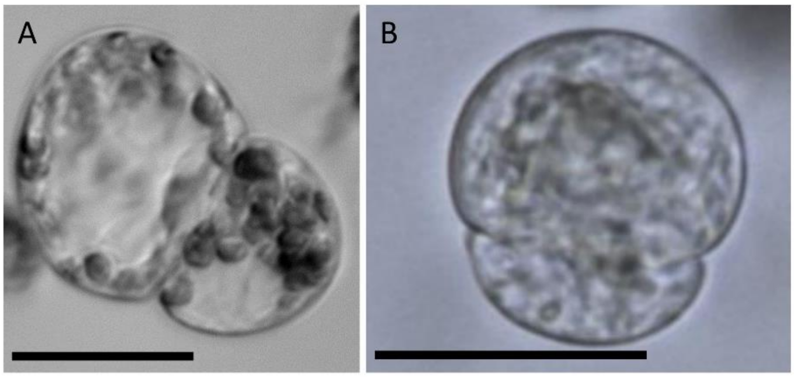

Figure S2. Example of two types of cell division: A- vacuolated; B- compact with de-differentiated chloroplasts. Scale bar- 20 μm.

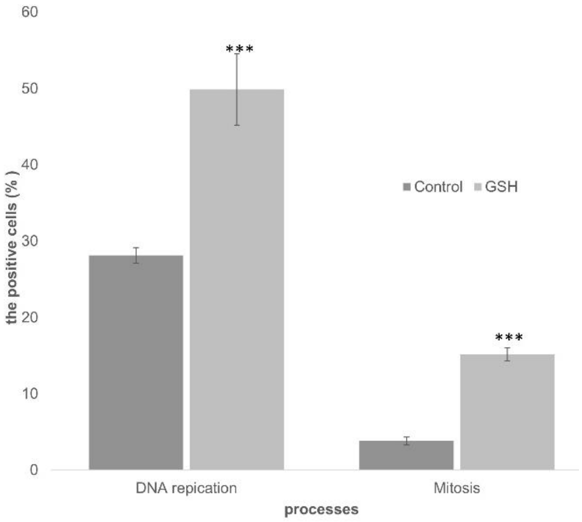

Figure S3. Effect of the GSH on DNA replication and cell proliferation. Cells were cultured on the TK30 medium supplemented with 2 μM 2,4-D or 2 μM 2,4-D + 1 mM GSH. DNA replication and cell proliferation ratio were counted after 84 hours of cultivation. % of positive cells means % of cell pass DNA replication or mitosis. (N=4). Error bar means standard error.

Publisher's Note: MDPI stays neutral with regard to jurisdictional claims in published maps and institutional affiliations.

Copyright: © 2021 by the authors. Submitted for possible open access publication under the terms and conditions of the Creative Commons Attribution (CC BY) license (http://creativecommons.org/licenses/by/4.0/).
